# Identification of a Novel Human Polyomavirus in Organs of the Gastrointestinal Tract

**DOI:** 10.1371/journal.pone.0058021

**Published:** 2013-03-13

**Authors:** Sarah Korup, Janita Rietscher, Sébastien Calvignac-Spencer, Franziska Trusch, Jörg Hofmann, Ugo Moens, Igor Sauer, Sebastian Voigt, Rosa Schmuck, Bernhard Ehlers

**Affiliations:** 1 Division of Viral Infections, Robert Koch Institute, Berlin, Germany; 2 Research Group Emerging Zoonoses, Robert Koch Institute, Berlin, Germany; 3 Institute of Virology, Charité - Universitätsmedizin Berlin, Berlin, Germany; 4 Department of Medical Biology, University of Tromsø, Tromsø, Norway; 5 General, Visceral, and Transplantation Surgery, Experimental Surgery and Regenerative Medicine, Charité-Campus Virchow, Charité Universitätsmedizin Berlin, Germany; University of Kansas Medical Center, United States of America

## Abstract

Polyomaviruses are small, non-enveloped viruses with a circular double-stranded DNA genome. Using a generic polyomavirus PCR targeting the VP1 major structural protein gene, a novel polyomavirus was initially identified in resected human liver tissue and provisionally named Human Polyomavirus 12 (HPyV12). Its 5033 bp genome is predicted to encode large and small T antigens and the 3 structural proteins VP1, VP2 and VP3. Phylogenetic analyses did not reveal a close relationship to any known human or animal polyomavirus. Investigation of organs, body fluids and excretions of diseased individuals and healthy subjects with both HPyV12-specific nested PCR and quantitative real-time PCR revealed additional virus-positive samples of resected liver, cecum and rectum tissues and a positive fecal sample. A capsomer-based IgG ELISA was established using the major capsid protein VP1 of HPyV12. Seroprevalences of 23% and 17%, respectively, were determined in sera from healthy adults and adolescents and a pediatric group of children. These data indicate that the virus naturally infects humans and that primary infection may already occur in childhood.

## Introduction

Polyomaviruses are small non-enveloped viruses with a circular double-stranded DNA genome. They usually cause asymptomatic primary infections, and persist in the body throughout life [1]. Seroprevalence studies have revealed that most primary infections occur in childhood and that the majority of healthy adults had contact to one or more polyomaviruses [2]–[5]. In immunocompromised individuals, polyomavirus reactivation can be associated with clinical disease, i.e., JCPyV with progressive multifocal leukoencephalopathy, BKPyV with nephropathy and cystitis (both reviewed in: [6], [7]), MCPyV with Merkel cell carcinoma [8], and TSPyV with *Trichodysplasia spinulosa* [9]. For the other human polyomaviruses known to date (KIPyV, WUPyV, HPyV6, HPyV7, HPyV9, MWPyV/HPyV10/MXPyV and STLPyV [10]–[17]) no associations with pathologic conditions have been found. BKPyV and JCPyV persist in the kidney [1], and MCPyV, HPyV6, HPyV7 and TSPyV are members of the skin microbiome and/or are detected in skin diseases ([8], [9], [12], [15], [18]; reviewed in: [19]). The tropism of the other human polyomaviruses is largely unknown.

Most human polyomaviruses were discovered by examining body fluids, se- and excretions [10]–[14], [16], [20]. In the present study we performed a search for yet unknown human polyomaviruses with generic PCR and focused on organs of the gastrointestinal tract since a number of human polyomaviruses had been detected in feces and sewage water [10], [16], [21]–[29]. In addition, spleens and lymph nodes were tested, since JCPyV, MCPyV, and MCPyV-related chimpanzee polyomaviruses had been reported to be present in these organs [30]–[32]. Of the known human polyomaviruses, only MCPyV and TSPyV were detected in the investigated organs. However, in resected liver, a novel polyomavirus was discovered.

## Methods

### Ethic statement

The study protocol for collection of gastrointestinal organ samples was approved by the local ethics committee of the Charité - Universitätsmedizin Berlin. Written informed consent was obtained from study participants. The ethics committee approved this consent procedure. Residual anonymized material from spleen and lymph node samples was collected from deceased individuals who donated organs for transplantation. Informed verbal consent for organ donation was obtained from the next of kin and documented by the responsible physician. The anonymized collection of residual materials was approved by the local ethics committee of the Charité - Universitätsmedizin Berlin. All samples of body fluids and excretions were residual materials from anonymized specimens originally submitted for routine diagnostics. Additionally, retain samples from anonymized blood donors collected after the retention period were used in this study. All procedures were in accordance with the ethical standards of the responsible committee on human experimentation and with the Helsinki Declaration.

### Sample collection

For PCR purposes, spleen (n = 61) and lymph node (n = 22) specimens were provided by the German Foundation for Organ Transplants (Deutsche Stiftung Organtransplantation, DSO), Frankfurt am Main, Germany. Liver (n = 124), gall bladder (n = 21), esophagus (n = 2), stomach (cardia; n = 2), colon (n = 4) and rectum specimens (n = 6) were collected at the clinic of general, visceral and transplantation surgery of the Charité, Berlin, Germany. Serum, plasma, urine, fecal, bronchoalveolar lavage and cerebrospinal fluid samples were taken from the panel that had been previously collected in Germany and analyzed for the presence of HPyV9 [11]. Oral fluids (n = 30) were collected from patients with suspected but not confirmed measles virus infection in Germany. Native tissue samples were kept frozen at −80°C and liquid samples at −20°C.

For ELISA, serum samples from healthy adolescents and adults (age 16 to 72 years; n = 299) and from pediatric patients (age 2–11 years; n = 74) were used that had been collected previously for the determination of HPyV9 seroprevalence [33].

### DNA extraction and PCR methods

DNA was extracted, purified and generic polyomavirus PCR was carried out as described previously [11], [32]. To obtain additional sequence information of the novel polyomavirus, a 950 bp genome fragment was amplified with nested PCR using two degenerate sense primers targeting the VP3 gene of polyomaviruses [11] and two virus-specific antisense primers derived from the novel VP1 sequence (primers listed in Table S1; PCR conditions in Table S2). From the resulting sequence, tail-to-tail primers were derived and used in nested long-distance (LD) PCR for the amplification and sequencing of the remaining part of the virus genome (Tables S1 and S2). For diagnostic detection of the novel polyomavirus, specific nested PCR primers were selected (Table S1) and used under the conditions described in Table S2.

For specific quantitative PCR (qPCR), targeting a 139 bp sequence of the VP1 gene, a sense primer (5′- GTGGGAAGCTGTCAGTGTGA), an antisense primer (5′- CCACCTACTGCAAACATGTG) and a TaqMan probe (*FAM*-ACTACAGGATGGCCTACCCCATTGTCAGTC-*TAMRA*) were selected. Five µl of DNA from fluid samples and 250 ng of DNA from tissue samples were analyzed in a 96-well plate format. The PCR was performed in a total volume of 25 µl with 2 U Platinum Taq DNA polymerase (Life Technologies, Darmstadt, Germany), 400 nM of each primer, 150 nM probe, 800 µM dNTP PCR Mix (Metabion, Martinsried, Germany) and 4.5 mM MgCl_2_. An MX 3000P (Stratagene, Waldbronn, Germany) was used with the following cycling conditions: 95°C for 5 min and 45 cycles of 95°C for 15 sec, followed by 59°C for 30 sec. Analysis was performed using the MXPro3000P V 4.10 software (Stratagene).

### Genome annotation

Open reading frames (ORFs) were predicted using Geneious Pro 5.5.7 software. The region encoding the large T and small T antigens (LTAg; STAg) was scanned for splice sites using MacVector 12 software. Conserved motifs in all ORFs were identified using the EMBOSS Needle-Pairwise Sequence Alignment (Rice et al., 2000). Putative binding sites of transcription factors were predicted using Alggen PROMO software [34], [35]. Amino acid percentage identities were calculated, and palindromes in the NCCR were identified using the EMBOSS Needle-Pairwise Sequence Alignment [36].

### Phylogenetic analysis

VP1, VP2 and LTAg protein alignments comprising representative sequences from all polyomaviruses currently recognized as species by the International Committee on Taxonomy of Viruses (ICTV; [37]) or recently reported to likely account for novel species (e.g., MWPyV; [10]) were computed and used for phylogenetic analyses (Table S3). These datasets notably included 10 non-human polyomaviruses whose genomes were recently sequenced in our laboratory and which will be the subject of a separate publication. Phylogenetic analyses were performed using a workflow described previously [32], which ended up with maximum likelihood (ML) and Bayesian tree reconstruction. The three coding sequences were processed and analyzed individually.

It should be noted that during the revision process of this manuscript, novel polyomaviruses were identified (e.g. STLPyV). None, however, was closely related to the novel human polyomavirus described in this study (data not shown). It is therefore not expected that their inclusion would affect its phylogenetic placement.

### Expression and purification of recombinant VP1 proteins

The major capsid proteins VP1 genes of HPyV12 and the avian polyomavirus APyV (former name: BFDPyV) were expressed as described previously [33]. In brief, the VP1 sequences were codon-optimized, commercially synthesized (MrGene GmbH, Regensburg, Germany) and inserted into a pTriEx-1.1 plasmid that generates VP1 constructs tagged with a 6x His-tag at the N-terminus. After transformation and expression in *E. coli* Rosetta(DE3)pLacITM cells (Novagen, San Diego, USA), insoluble recombinant proteins were obtained in inclusion bodies and purified with BugBuster Protein Extraction Reagent (Novagen) after lysis of cells and inclusion bodies with rLysozyme™ (Novagen). Purification of VP1 from other *E. coli* proteins was done under denaturing conditions with 8 M urea (Roth, Karlsruhe, Germany) using HIS-Select® Nickel Affinity Gel (Sigma-Aldrich, St. Louis, USA). Native conformation of the VP1 proteins was reconstituted by removing urea by dialysis. Purity of proteins was analyzed with SDS–PAGE and Western Blot using an anti-His monoclonal antibody (Sigma-Aldrich, St. Louis, USA). Protein concentration was determined with a Pierce BCA Protein Assay Kit (Thermo Scientific, Rockford, USA).

### ELISA and statistical analysis

To detect antibodies with reactivity to HPyV12 VP1, an ELISA was performed as described earlier [33]. F96 maxisorp immuno plates (Nunc, Roskilde, Denmark) were incubated with purified VP1 (50 ng per well) in PBS (pH 7.2) for 1 h at 37°C. Plates were washed 3x with 800 µl PBS/0.05% Tween (PBS-T). To inhibit non-specific binding 200 µl blocking buffer (PBS-T with 5% milk powder) per well was added and incubated for 2 h at 37°C. Human sera were diluted 1∶200 and allowed to react with the antigen-coated wells for 1 h at 37°C. Plates were washed 3x with 800 µl PBS-T and a HRPO-conjugated, secondary rabbit anti-human IgG antibody (Dianova, Hamburg, Germany), diluted 1∶10,000, was added to detect IgG antibodies. After an additional washing step (3x with 800 µl PBS-T), peroxidase substrate TMB (tetramethylbenzidene, Taastrup, Denmark) was added for 10 min at room temperature in the dark. The reactions were stopped with 2 N H_2_SO_4_. Optical density was measured on a microplate spectrometer (BMG Labtech, Offenburg, Germany) at λ = 450 nm. All blank wells had absorbance values<0.1. The data were analyzed with the Χ2-test to estimate significance of differences among independent groups of individuals. For each ELISA plate, a fixed set of sera was used to control for interserial variations. The cut-off value (COV) for the ELISA was determined experimentally. The background reactivities detected in wells without antigen coating and those without both antigen and serum (blanks) were subtracted from the ODs measured in VP1-coated wells. The COV defining a positive serologic response was defined as the mean of all negative ODs plus standard deviation (COV HPyV12: OD_450_ = 0.086). To further ensure that the final OD_450_ values for HPyV12 VP1 were not in part derived from unspecific antibody binding, reactivity of the sera to VP1 of an avian polyomavirus (APyV) was measured (Mean OD_450_ = 0.07), and the values obtained for each serum were subtracted from the ODs measured for HPyV12 VP1.

### Nucleotides sequence accession numbers

The annotated, complete genome sequence of HPyV12 has been submitted to GenBank (accession number JX308829). The Genbank accession numbers of PyV genomes that were used in phylogenetic analysis are listed in Table S3.

## Results

### Identification of HPyV12 and complete genome sequencing

Organs of the gastrointestinal tract (liver, gall bladder, esophagus, stomach, colon, rectum; n = 159) as well as spleen (n = 61) and lymph node (n = 22) specimens were tested with generic polyomavirus PCR targeting the VP1 gene (Table 1). In 13/242 PCR assays, fragments of the expected size were obtained, purified and sequenced. BLAST analysis [38] revealed that 8 of the 13 sequences originated from MCPyV (detected in 3 livers, 3 spleens, and 2 lymph nodes) and 1 from TSPyV (detected in a lymph node). Most importantly, an unknown polyomavirus sequence was amplified from liver specimens of 4 individuals, revealing a relatively low level of identity to all human and nonhuman polyomaviruses (pairwise amino acid identities: 51–67%). The complete genome sequence was then generated from a liver specimen with VP3/VP1-PCR and LD-PCR as described in the *Methods* section. After sequencing all products, a final circular genome of 5033 bp was obtained. Since 11 phylogenetically distinct polyomaviruses of human origin are known at present, the virus from which the sequence originated was tentatively named Human Polyomavirus 12 (HPyV12).

**Table 1:** Detection of polyomaviruses in gastrointestinal and lymphoid organs with generic PCR.

Sample type	Source	No. of samples tested	Polyomaviruses^a^ (no. of specimens)
Gastro-intestinal tract	Patients with malignant diseases	159	
Liver		124	HPyV12 (4), MCPyV (3)
Gall bladder		21	(0)
Esophagus		2	(0)
Stomach (Cardia)		2	(0)
Colon		4	(0)
Rectum		6	(0)
Spleen	Donors for transplantation	61	MCPyV (3)
Lymph node	Donors for transplantation	22	MCPyV (2), TSPyV (1)
Σ		242	(13)

a Detected with generic PCR.

### Analysis of the HPyV12 genome

**Figure 1:**
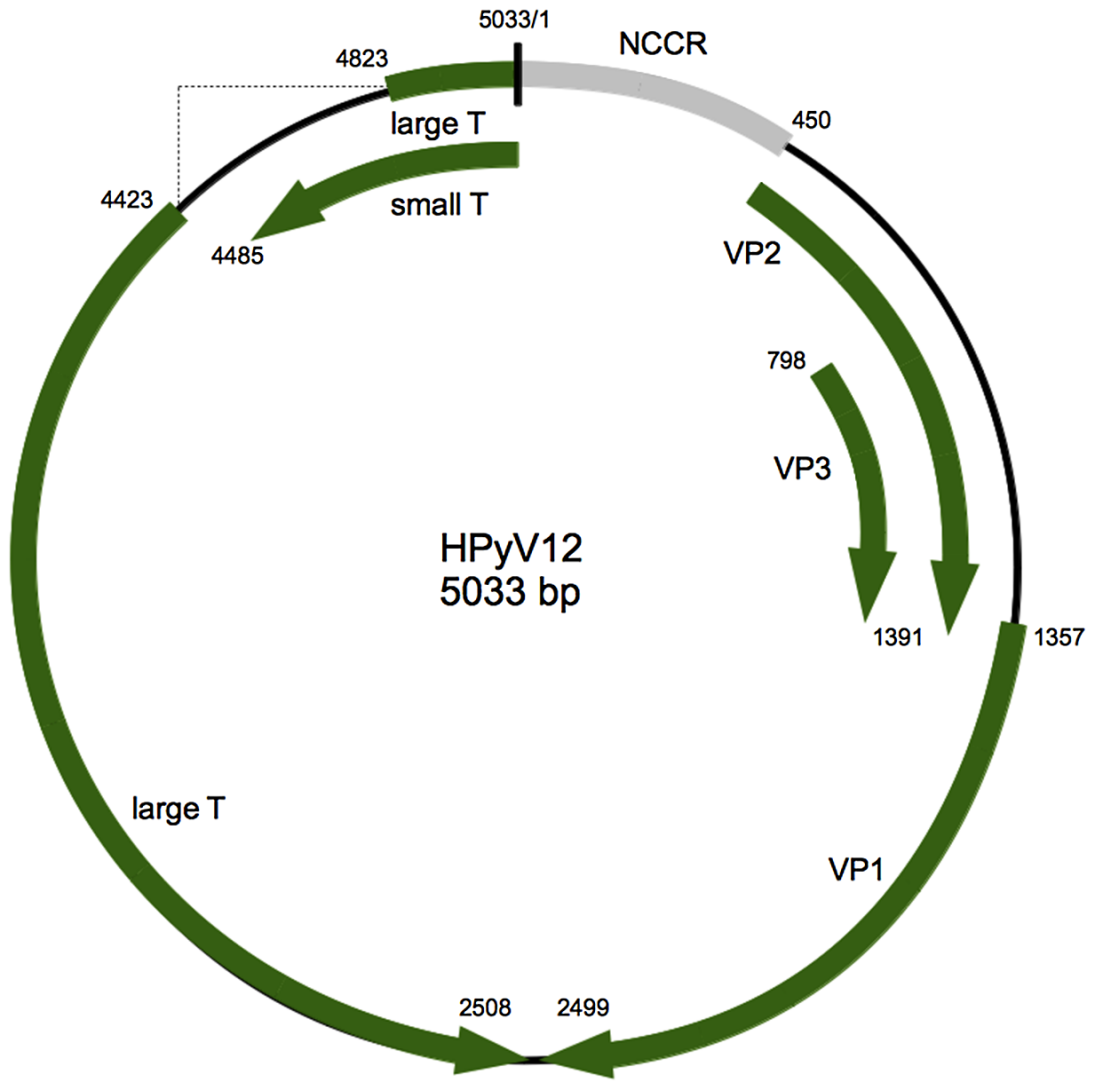
Genome organization of HPyV12. Putative coding regions for VP1 to VP3, STAg antigen, and LTAg antigen are marked by arrows.

Analysis of the HPyV12 genome for putative open reading frames (ORFs) revealed a genome structure typical for polyomaviruses. It includes an early region encoding regulatory proteins (STAg and LTAg) and a late region encoding structural proteins (VP1, VP2 and VP3) that are separated by a non-coding control region (NCCR) (Figure 1). An ORF encoding for the auxiliary agnoprotein [39] was not identified. The ORF locations on the viral genome, the encoded proteins and their percentages of identity to other human polyomaviruses are listed in Table 2.

**Table 2:** Putative proteins encoded by HPyV12 and amino acid identities between HPyV12 and other polyomaviruses.

			Amino acid sequence identity (%)^a^						
Protein	Putative coding region	Amino acids^b^	TSPyV^c^	MCPyV	BKPyV	HPyV9	BatPyV	OraPyV	GHPyV
VP1	1357∶2499	380	60	57	54	59	61	63	62
VP2	450∶1391	313	42	31	31	36	36	42	35
VP3	798∶1391	197	40	24	28	37	37	38	32
ST Ag	5033–4485	182	30	34	30	33	29	30	26
LTAg exon 1	5033–4823	708	46	52	38	41	39	46	31
LTAg exon 2	4423–2508								

a Determined with blastX (pairwise).

b Number of amino acids.

c Accession numbers for TSPyV to GHPyV: GU989205; HM355825; NC_001538; NC_015150; JQ958892; FN356901; NC_004800.

HPyV12 LTAg and STAg share 71 amino acid residues that are encoded at the N-terminus. This region contains the DnaJ domain HPDKGG which is fully conserved between all known human polyomaviruses identified so far [40]. However, HPyV12 LTAg lacks the retinoblastoma binding motif LxCxE which is present in LTAg sequences of all known human polyomaviruses. There is a putative ATPase domain in the C-terminal part of the protein. This domain consists of the conserved GPxxxGKT (GPINSGKT; residues 498–505 in HPyV12 LTAg) and GxxxVNLE (GSVTVNLE; residues 576–583 in HPyV12 LTAg). HPyV12 LTAg also has a Lys-rich sequence (PKSKKAK; residues 205–211) which may act as a nuclear localization signal. A Cys-rich sequence (Cx_7_CxCX_2_Cx_22_CxCx_2_Cx_3_WFG) which has been shown to be required for binding of protein phosphatase PP2A by STAgs of other polyomaviruses, is conserved in HPyV12 STAg (residues 100–147). The NCCR contains 4 repeats of the putative GAGGC LTAg binding site and AT-rich palindromic sequences. The NCCR possesses potential binding sites for numerous cellular transcription factors, but the functional importance of these sites remains to be proven (Figure S4).

### Phylogenetic analysis of HPyV12

**Figure 2:**
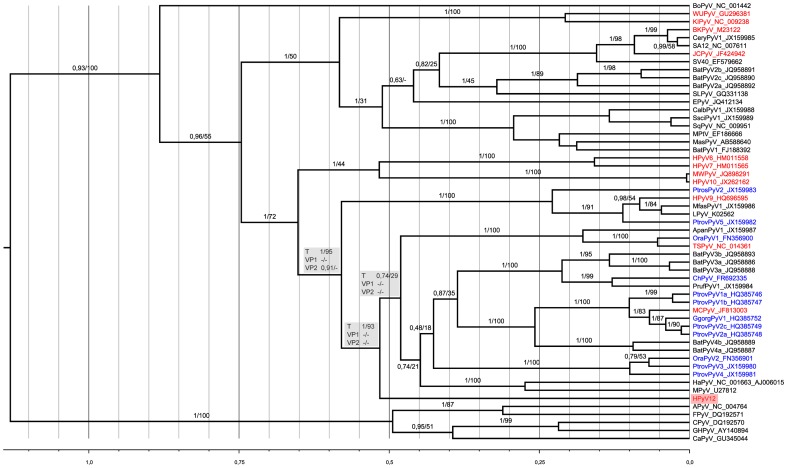
Phylogenetic analysis of HPyV12. A Bayesian chronogram was deduced from the analysis of a 488 amino acid alignment of LTAg sequences. Polyomaviruses identified from human hosts are in red, from apes in blue. The human polyomaviruses MXPyV and the US strain of MWPyV have the same phylogenetic position as HPyV10 and are not shown. The sequence of the very recently discovered human STLPyV which is most closely related to HPyV10, MWPyV and MXPyV, became only available at the end of the revision process of this manuscript; it was therefore not included in the datasets put together for the present study. Statistical support for branches is given as posterior probability/bootstrap. For three branches defining potentially meaningful bipartitions (with respect to the question of the phylogenetic placement of HPyV12), statistical support observed for the corresponding bipartitions in the VP2 and VP1 analyses is also shown (grey panels). Hyphen indicates that bipartition was not observed in the ML or Bayesian tree. The scale axis is in amino acid substitution per site. This chronogram was rooted using a relaxed clock. A maximum likelihood analysis of the same dataset concluded to a similar topology and is thus not shown here.

Three alignments consisting of 98, 259, and 488 amino acids were generated from HPyV12 VP2, VP1 and LTAg sequences, respectively, and those of other polyomaviruses for which complete genomes were available (Table S3). On this basis, ML and Bayesian analyses of the individual coding sequences were performed. Only the analyses of LTAg sequences did support a clear placement of HPyV12, which was neither contradicted nor supported by VP2 and VP1 analyses (Figure 2). In the LTAg phylogeny, HPyV12 appeared as the earliest offshoot of a large clade comprising (by decreasing order of frequency) ape, bat, monkey, rodent and human PyVs (i.e., MCPyV and TSPyV). The branching order of the main lineages comprised within this clade could not be determined as most internal branches only received moderate statistical support. On the other hand, the clade comprising HPyV12 appeared quite clearly as the sister clade to a group of primate polyomaviruses including HPyV9.

### Prevalence of HPyV12 in clinical samples

The 242 samples from gastrointestinal organs, spleens and lymph nodes were re-evaluated with diagnostic nested PCR and qPCR. The presence of HPyV12 sequences was confirmed in the samples that were originally HPyV12-positive in the initial generic PCR. In addition, 10 liver samples tested positive that had been negative in the generic PCR. In total, 14/124 liver samples (11%) were HPyV12-positive (Table 3). One positive sample each was also identified among rectum (n = 6) and colon (n = 4) samples. Analysis of gall bladder (n = 21), esophagus and stomach (cardia) (each n = 2) yielded negative results (Table 3). Spleens (n = 61) and lymph nodes (n = 22) were also HPyV12-negative (Table 3). To further elucidate the prevalence of HPyV12, body fluids and excretions were analyzed. Testing of feces (n = 56) revealed one HPyV12-positive sample. Plasma (n = 54), serum (n = 45), urine (n = 152), oral fluids (n = 30), bronchoalveolar lavage fluids (n = 22) and cerebrospinal fluids (n = 35) were negative (Table 3). In qPCR, the HPyV12-positive DNA samples revealed genome copy numbers of up to 133/PCR reaction (equivalent to 27 copies/µl DNA from fluid samples; 532 copies/ µg DNA from tissue samples). In summary, analysis of 636 clinical samples revealed the presence of HPyV12 only in organs of the gastrointestinal tract and in feces.

**Table 3:** Detection of HPyV12 with PCR.

Sample type	Source(s)	No. of samples tested	No. of samples PCR-positive for HPyV12^e^
Gastro-intestinal tract	Patients with malignant diseases	159	16
Liver		124	14
Gall bladder		21	0
Esophagus		2	0
Stomach (Cardia)		2	0
Colon		4	1
Rectum		6	1
Lymph node	Donors for transplantation	22	0
Spleen	Donors for transplantation	61	0
Feces	Patients with diarrhea	56	1
Plasma	Kidney transplant recipients^a^	54	0
Serum	Liver transplant recipients	45	0
Urine	Kidney transplant recipients^a^	12	0
	Stem cell transplant recipients^a^	14	0
	Patients with multiple sclerosis^b^	9	0
	Immunocompromised patients with malignant disease^a^	76	0
	Other^a^	41	0
			
Oral fluid	From measles diagnostics	30	0
Bronchoalveolarlavage fluid	Patients with pneumonia^c^	22	0
Cerebrospinal fluid	Patients with leukoencephalopathy^d^	35	0
Σ		636	17

a Samples collected for BKPyV infection diagnosis.

b From patients under natalizumab (Tysabri) therapy.

c Samples collected for herpesvirus infection diagnosis.

d Samples collected for JCPyV infection diagnosis.

e Combined results of specific nested PCR and real-time PCR; samples were considered as positive if the result could be independently reproduced on different days.

### Seroprevalence of HPyV12

**Figure 3:**
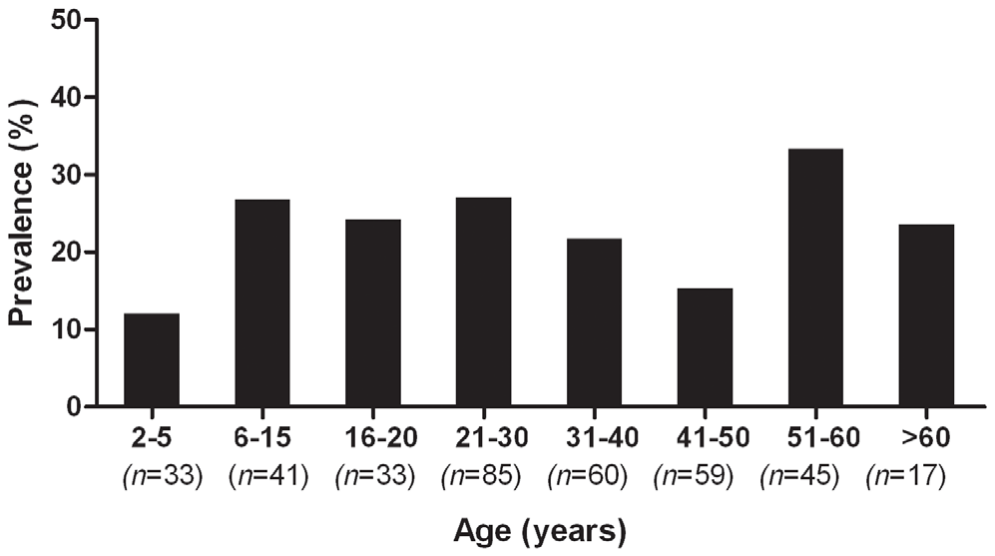
Reactivity of human sera to VP1 of HPyV12. The percentage of seroreactivity of pediatric sera (n = 74; 2–11 years) and sera of healthy adolescents and adults (n = 299; age: 16–72 years) in ELISA is shown. The results were stratified by age.

To detect antibodies against HPyV12 VP1, a capsomer-based ELISA was performed. A pediatric population of 74 subjects and 299 healthy adults and adolescents were tested, and the data were stratified by age. The seroprevalence of HPyV12 was 12% in children of age 2–5 and rose to 26% in the group of age 6–11. In young adults of age 21–30, a prevalence of 27% was determined. In older adults the prevalence ranged between 15% and 33% (Figure 3). A difference in HPyV12 seroprevalence between male and female adults was not observed (data not shown).

## Discussion

The present study reports on the discovery of a hitherto unknown polyomavirus (HPyV12) in humans. The novel polyomavirus was detected by generic PCR, real-time PCR and conventional nested PCR in liver specimens of 14 individuals as well as in the colon, rectum and feces of single individuals (Tables 1 and 2). To study the seroprevalence of HPyV12, ELISA was performed that was considered to specifically detect VP1 antibodies against HPyV12. The assay revealed that healthy individuals are frequently infected with HPyV12 before the age of twenty (Figure 3). We believe that cross-reaction with antibodies raised against other known human polyomaviruses is unlikely to explain these results as none of their VP1 proteins displays more than 60% identity to HPyV12 VP1. Cross-reactions of VP1 proteins in serological assays have only been observed when the proteins revealed more than 75% identity [2], [4], [5], [11], [41], and no cross-reactivity between VP1 antibodies from the known human polyomaviruses has been detected [2], [4], [5], [11], [41]–[44]. We cannot, however, exclude cross-reactivity between HPyV12 VP1 and antibodies directed against not yet identified human polyomaviruses. The seroprevalence reported here should therefore be regarded as a first estimate. Taken together, HPyV12 can be regarded as a virus that naturally infects humans at young age and resides in the gastrointestinal tract.

It is remarkable that - besides the novel HPyV12 - only MCPyV was detected in liver and other organs of the gastrointestinal tract. It is unlikely that this would be the result of a failure of the applied PCR system to detect other polyomaviruses since this system allowed for the recovery of a number of polyomaviruses across the entire phylogeny [11], [32]. Therefore, this may indicate that the other human polyomaviruses were at least not present in high copy numbers in the gastrointestinal tract. The multiple detection of MCPyV in livers is in line with a previous study that reported detection of MCPyV in livers and other sites of the gastrointestinal tract [25]. Since liver specimens were highly overrepresented in our panel of gastrointestinal tract specimens (124/159), it could be concluded that polyomaviruses other than HPyV12 and MCPyV do not reside regularly in the liver. A prediction on the other organs of the gastrointestinal tract studied here (gall bladder, stomach, colon, rectum) was not possible since sample numbers were too small. A similarly low abundance of polyomaviruses was observed in lymphoid organs (Table 1). This is in contrast to the frequent presence of polyomaviruses in lymphoid organs of the closest relative of humans, the chimpanzee [32], and indicates a major difference in polyomavirus tropism between these closely related hominine hosts.

Human polyomaviruses, such as BKPyV and JCPyV, induce tumors in animal models. However, their role in human cancer remains a matter of debate [45]-[47]. The recently identified MCPyV is the first polyomavirus that is etiologically associated with a human tumor [8], [19], [48]. It is noteworthy that HPyV12 is the first human polyomavirus whose LTAg lacks any LxCxE motif. As this motif is essential to the retinoblastoma protein-dependent transforming activity of other polyomaviruses, HPyV12 may exhibit a reduced transforming potential. It is clear that, in addition to continued efforts aimed at completing our view of the diversity of the human polyomavirome, efforts should also be made to characterize the biology and the possible impact on human health of novel human polyomaviruses.

## Supporting Information

Figure S1 **Putative transcription factor binding sites in the non-coding control region of HPyV12.** The numbers in the upper boxes (10 to 450) refer to the nucleotide position in the NCCR, while the color-shaded numbers refer to the particular transcription factor that binds. The ALGGEN PROMO algorithm was used.
(TIF)

Table S1 **Primers for amplification of HPyV12.**
(DOCX)

Table S2 **PCR conditions for amplification of HPyV12.**
(DOCX)

Table S3 **Published polyomaviruses used in phylogenetic analysis.**
(DOCX)
