# Supplementary figures and images for: Identification of a Novel Human Polyomavirus in Organs of the Gastrointestinal Tract
Source: PLoS One. 2013 Mar 13;8(3):e58021. doi: 10.1371/journal.pone.0058021 (PMC3596337; doi:10.1371/journal.pone.0058021)

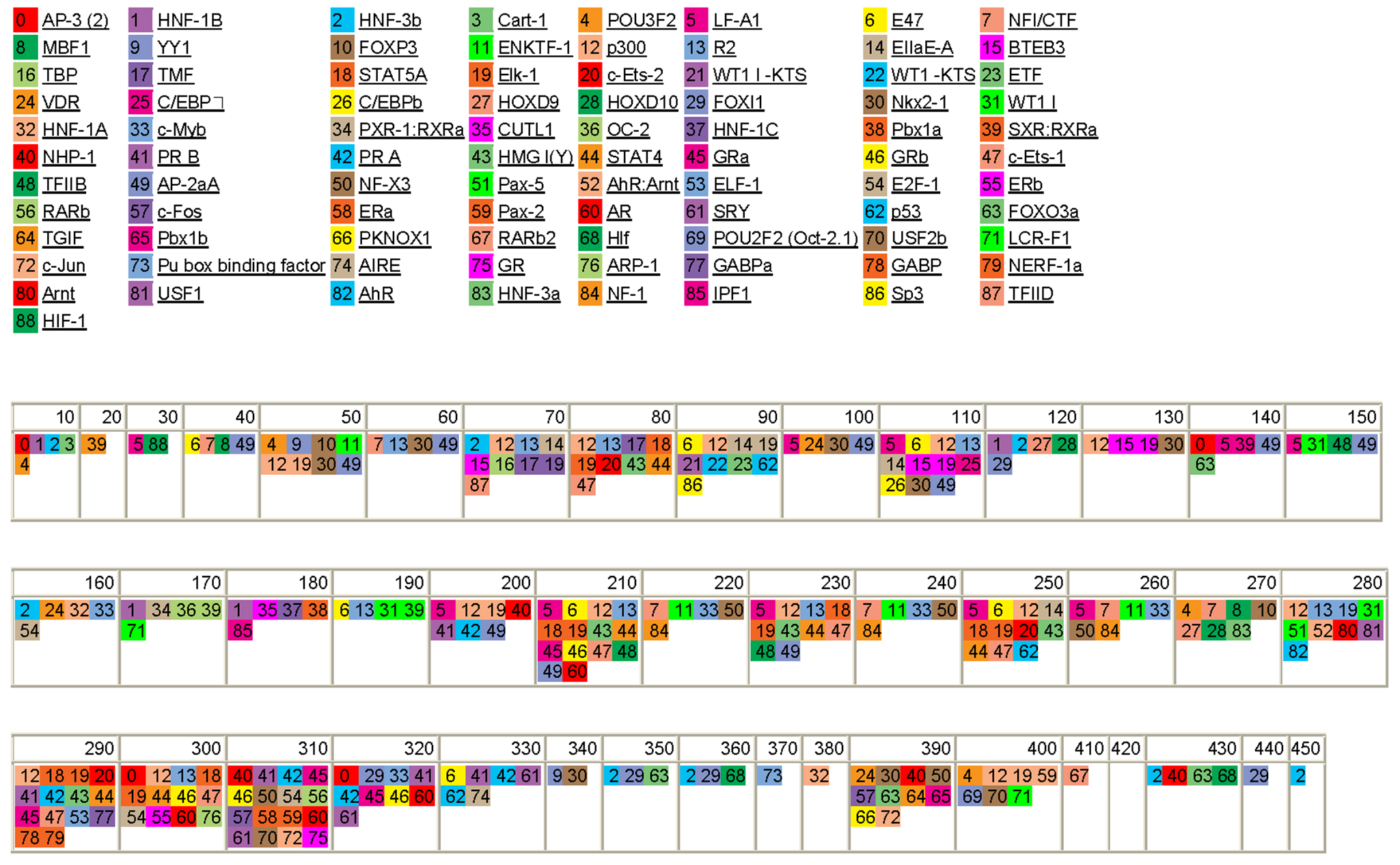

Supplement: Figure S1 — Putative transcription factor binding sites in the non-coding control region of HPyV12. The numbers in the upper boxes (10 to 450) refer to the nucleotide position in the NCCR, while the color-shaded numbers refer to the particular transcription factor that binds. The ALGGEN PROMO algorithm was used. (TIF) [file pone.0058021.s001.tif]
