# Supplementary material for: Identification of a Novel Human Polyomavirus in Organs of the Gastrointestinal Tract
Source: PLoS One. 2013 Mar 13;8(3):e58021. doi: 10.1371/journal.pone.0058021 (PMC3596337; doi:10.1371/journal.pone.0058021)
Supplement: Table S1 — Primers for amplification of HPyV12. (DOCX) [file pone.0058021.s002.docx]

**Table S1 Primers for amplification of HPyV12**

| **Primer** | **PCR round** | **Target** | **Sequence (5´-3´)** | **Genome position** | **Fragment**  **size (bp)** |
| --- | --- | --- | --- | --- | --- |
| **Diagnostic PCR** |  |  |  |  |  |
| HPyV12-D-1 sense | 1 | VP1 | GGATGGACTTTTTCTCTCTTGTTGT | 2178:2202 | 189 |
| HPyV12-D-1 antisense |  | VP1 | CCTGACATCCGGGGCATCATA | 2346:2366 |  |
| HPyV12-D-2 sense | 2 | VP1 | TCTCTCTTGTTGTGATGTAATGGGA | 2190:2214 | 167 |
| HPyV12-D-2 antisense |  | VP1 | GGGGCATCATATTAGTAAACAAACT | 2332:2356 |  |
| **VP3/VP1 PCR** |  |  |  |  |  |
| PyV-VP3-1 sense | 1 | VP3 | AGAGTAACTCAAGACTGGAT(N^a^/I^b^)CT | 1242:1263 | 1021 |
| HPyV12-VP1-1 antisense |  | VP1 | TGGTAGGCCTCTAAATCTCTGATG | 2239:2262 |  |
| PyV-VP3-2 sense | 2 | VP3 | ACTCAAGACTGGATGCT(N^a^/I^b^)CCT | 1248:1267 | 1001 |
| HPyV12-VP1-2 antisense |  | VP1 | ATCTCTGATGGGTCCCACTCCCT | 2226:2248 |  |
| **Long-distance PCR** |  |  |  |  |  |
| HPyV12-LD-1 sense | 1 | VP1 | TCACAGCAGGGAGTGGGACCCA | 2219:2240 | 4456 |
| HPyV12-LD-1 antisense |  | VP1 | GCGATCAGTTTCCCTGGCAGTAGCT | 1617:1641 |  |
| HPyV12-LD-2 sense | 2 | VP1 | TGAGGAAAAGGGCTGTAAGAAATCC | 2282:2306 | 4313 |
| HPyV12-LD-2 antisense |  | VP1 | TCCGGGGATTTAGAAAGGCCTCAA | 1538:1561 |  |

^a^any base; ^b^inosine
