# Supplementary material for: Identification of a Novel Human Polyomavirus in Organs of the Gastrointestinal Tract
Source: PLoS One. 2013 Mar 13;8(3):e58021. doi: 10.1371/journal.pone.0058021 (PMC3596337; doi:10.1371/journal.pone.0058021)
Supplement: Table S2 — PCR conditions for amplification of HPyV12. (DOCX) [file pone.0058021.s003.docx]

**Table S2 PCR conditions for amplification of HPyV12**

| **Primer** | **PCR round** | **Total volume (µl)** | **Cycles** | **Cycling conditions** | **Reaction setup** |
| --- | --- | --- | --- | --- | --- |
| **Diagnostic PCR** | 1 | 25 | 1 | 95^o^C for 12 min | 2 units AmpliTaq Gold (Applied Biosystems) |
|  |  |  | 45 | 95^o^C for 30 sec | 20 pmol of each primer |
|  |  |  |  | 59^o^C for 30 sec | 200 µM dNTPs |
|  |  |  |  | 72^o^C for 2 min | 2 mM MgCl_2_ |
|  |  |  | 1 | 72^o^C for 15 min | 5% DMSO |
|  |  |  |  |  | sterilized distilled water up to 25µl |
|  | 2 | 25 |  | conditions as in round 1 | setup as in round 1 |
|  |  |  |  |  |  |
| **VP3/VP1-PCR** | 1 | 25 | 1 | 95^o^C for 12 min | 2 units AmpliTaq Gold (Applied Biosystems) |
|  |  |  | 45 | 95^o^C for 30 sec | 20 pmol of each primer |
|  |  |  |  | 50^o^C for 30 sec | 200 µM dNTPs |
|  |  |  |  | 72^o^C for 2 min | 2 mM MgCl_2_ |
|  |  |  | 1 | 72^o^C for 15 min | 5% DMSO |
|  |  |  |  |  | sterilized distilled water up to 25 µl |
|  | 2 | 25 |  | conditions as in round 1 | setup as in round 1 |

**……. Table S2 continued**

| **Long-distance PCR** | 1 | 50 | 1 | 94^o^C for 5 min | 2 units TaKaRa Ex Taq (Takara Bio inc.) |
| --- | --- | --- | --- | --- | --- |
|  |  |  | 15 | 98^o^C for 20 sec | 1x ExTaq buffer with MgCl_2_ (20 mM) |
|  |  |  |  | 64^o^C for 30 sec | 400 nM of each primer |
|  |  |  |  | 68^o^C for 7 min | 400 µM dNTPs |
|  |  |  | 15 | 98^o^C for 20 sec | sterilized distilled water up to 50 µl |
|  |  |  |  | 64^o^C for 30 sec |  |
|  |  |  |  | 68^o^C for 7 min + 5 sec |  |
|  |  |  | 1 | 72^o^C for 30 min |  |
|  | 2 | 50 | 1 | 94^o^C for 5 min | setup as in round 1 |
|  |  |  | 15 | 98^o^C for 20 sec |  |
|  |  |  |  | 60^o^C for 30 sec |  |
|  |  |  |  | 68^o^C for 7 min |  |
|  |  |  | 15 | 98^o^C for 20 sec |  |
|  |  |  |  | 60^o^C for 30 sec |  |
|  |  |  |  | 68^o^C for 7 min + 5 sec |  |
|  |  |  | 1 | 72^o^C for 30 min |  |
