# Supplementary material for: Identification of a Novel Human Polyomavirus in Organs of the Gastrointestinal Tract
Source: PLoS One. 2013 Mar 13;8(3):e58021. doi: 10.1371/journal.pone.0058021 (PMC3596337; doi:10.1371/journal.pone.0058021)
Supplement: Table S3 — Published polyomaviruses used in phylogenetic analysis. (DOCX) [file pone.0058021.s004.docx]

**Table S3 Published polyomaviruses used in phylogenetic analysis**

| **Virus species** | **Virus abbreviation** | **Host species** | **GenBank accession number** |
| --- | --- | --- | --- |
| Ateles paniscus polyomavirus 1 | ApanPyV1 | Red-faced spider monkey | JX159987 |
| Avian polyomavirus | APyV^a^ | Parrot and other bird species | NC_004764 |
| Baboon polyomavirus 1 | SA12 | Vervet monkey; Chacma baboon | [NC_007611](http://www.ncbi.nlm.nih.gov/sites/entrez?Db=genome&Cmd=ShowDetailView&TermToSearch=23927) |
| Bat polyomavirus 1 | BatPyV1 | North American Bat | [FJ188392](http://www.ncbi.nlm.nih.gov/sites/entrez?Db=genome&Cmd=ShowDetailView&TermToSearch=22960) |
| Bat polyomavirus 2a | BatPyV2a | South American Bat | JQ958892 |
| Bat polyomavirus 2b | BatPyV2b | .. | JQ958891 |
| Bat polyomavirus 2c | BatPyV2c | .. | JQ958890 |
| Bat polyomavirus 3a A1055 | BatPyV3a | .. | JQ958886 |
| Bat polyomavirus 3a B0454 | BatPyV3a | .. | JQ958888 |
| Bat polyomavirus 3b | BatPyV3b | .. | JQ958893 |
| Bat polyomavirus 4a | BatPyV4a | .. | JQ958887 |
| Bat polyomavirus 4b | BatPyV4b | .. | JQ958889 |
| B-lymphotropic polyomavirus | LPyV | African green monkey | [K02562](http://www.ncbi.nlm.nih.gov/sites/entrez?Db=genome&Cmd=ShowDetailView&TermToSearch=17050) |
| BK polyomavirus | BKPyV | Human | M23122 |
| Bornean orangutan polyomavirus | OraPyV1 | Orangutan | FN356900 |
| Bovine polyomavirus | BoPyV | Cattle | [NC_001442](http://www.ncbi.nlm.nih.gov/sites/entrez?Db=genome&Cmd=ShowDetailView&TermToSearch=10168) |
| Canary polyomavirus | CaPyV | Canary bird | GU_345044 |
| Cebus albifrons polyomavirus 1 | CalbPyV1 | White-fronted capuchin | JX159988 |
| Cercopithecus erythrotis polyomavirus 1 | CeryPyV1 | Red-eared guenon | JX159985 |
| Crow polyomavirus | CPyV | Jackdaw | DQ192570 |
| Chimpanzee polyomavirus | ChPyV | Central Chimpanzee | FR692335 |
| Horse polyomavirus | EPyV | Horse | JQ412134 |
| Finch polyomavirus | FPyV | Bullfinch | DQ192571 |
| Gorilla gorilla gorilla polyomavirus 1 | GgorgPyV1^b^ | Gorilla | HQ385752 |
| Goose hemorrhagic polyomavirus | GHPyV | Goose | AY140894 |
| Hamster polyomavirus | HaPyV | Hamster | NC_001663; AJ006015 |

**… Table S3 continued**

| **Virus species** | **Virus abbreviation** | **Host species** | **GenBank accession number** |  |
| --- | --- | --- | --- | --- |
| Human polyomavirus 6 | HPyV6 | Human | HM011558 | |
| Human polyomavirus 7 | HPyV7 | .. | HM011565 | |
| Human polyomavirus 9 | HPyV9 | .. | HQ696595 | |
| Human polyomavirus 10 | HPyV10 | .. | JX262162 | |
| JC polyomavirus | JCPyV | .. | JF424942 | |
| KI polyomavirus | KIPyV | .. | NC_009238 | |
| Macaca fascicularis polyomavirus 1 | MfasPyV1 | Crab-eating macaque | JX159986 | |
| Mastomys polyomavirus | MasPyV | Mouse | AB_588640 | |
| Merkel cell polyomavirus | MCPyV | Human | JF813003 |  |
| Murine pneumotropic polyomavirus | MPtV | Mouse | EF186666 |  |
| Murine polyomavirus | MPyV | .. | U27812 |  |
| MW polyomavirus | MWPyV | Human | JQ898291 |  |
| Pan troglodytes schweinfurthii polyomavirus 2 | PtrosPyV2 | Eastern Chimpanzee | JX159983 |  |
| Pan troglodytes verus polyomavirus 1a | PtrovPyV1a^b^ | Western Chimpanzee | HQ385746 |  |
| Pan troglodytes verus polyomavirus 1b | PtrovPyV1b^b^ | .. | HQ385747 |  |
| Pan troglodytes verus polyomavirus 2a | PtrovPyV2a^b^ | .. | HQ385748 |  |
| Pan troglodytes verus polyomavirus 2c | PtrovPyV2c^b^ | .. | HQ385749 |  |
| Pan troglodytes verus polyomavirus 3 | PtrovPyV3 | .. | JX159980 |  |
| Pan troglodytes verus polyomavirus 4 | PtrovPyV4 | .. | JX159981 |  |
| Pan troglodytes verus polyomavirus 5 | PtrovPyV5 | .. | JX159982 |  |
| Piliocolobus rufomitratus polyomavirus 1 | PrufPyV1 | Eastern red colobus | JX159984 |  |
| Saimiri sciureus polyomavirus 1 | SsciPyV1 | Squirrel monkey | JX159989 |  |
| Simian virus 40 | SV40 | Rhesus monkey | EF579662 |  |
| Sea lion polyomavirus 1 | SLPyV | Sea lion | GQ331138 |  |
| Squirrel monkey polyomavirus | SqPyV | Squirrel monkey | [NC_009951](http://www.ncbi.nlm.nih.gov/sites/entrez?Db=genome&Cmd=ShowDetailView&TermToSearch=21537) |  |
| Sumatran orangutan polyomavirus | OraPyV2 | Orangutan | FN356901 |  |
| Trichodysplasia spinulosa-associated polyomavirus | TSPyV | Human | [NC_014361](http://www.ncbi.nlm.nih.gov/sites/entrez?Db=genome&Cmd=ShowDetailView&TermToSearch=21537) |  |
| WU polyomavirus | WUPyV | .. | GU296381 |  |

^a^ Former name: BFDPyV

^b^ Abbreviation in original reference [Leendertz et al. (2011) J Virol 85: 916-924] : GggPyV1 (GgorgPyV1), PtsPyV1 (PtrosPyV1), PtvPyV1a (PtrovPyV1a), PtvPyV1b (PtrovPyV1b), PtvPyV2a (PtrovPyV2a), PtvPyV2b (PtrovPyV2b), PtvPyV2c (PtrovPyV2c)
